# Role and mechanisms of autophagy, ferroptosis, and pyroptosis in sepsis-induced acute lung injury

**DOI:** 10.3389/fphar.2024.1415145

**Published:** 2024-08-05

**Authors:** Yao Shen, Yingying He, Ying Pan, Li Liu, Yulin Liu, Jing Jia

**Affiliations:** ^1^ Department of Anesthesiology, The Affiliated Hospital, Southwest Medical University, Luzhou, China; ^2^ Anesthesiology and Critical Care Medicine Key Laboratory of Luzhou, The Affiliated Hospital, Southwest Medical University, Luzhou, China

**Keywords:** sepsis, acute lung injury (ALI), autophagy, ferroptosis, pyroptosis

## Abstract

Sepsis-induced acute lung injury (ALI) is a major cause of death among patients with sepsis in intensive care units. By analyzing a model of sepsis-induced ALI using lipopolysaccharide (LPS) and cecal ligation and puncture (CLP), treatment methods and strategies to protect against ALI were discussed, which could provide an experimental basis for the clinical treatment of sepsis-induced ALI. Recent studies have found that an imbalance in autophagy, ferroptosis, and pyroptosis is a key mechanism that triggers sepsis-induced ALI, and regulating these death mechanisms can improve lung injuries caused by LPS or CLP. This article summarized and reviewed the mechanisms and regulatory networks of autophagy, ferroptosis, and pyroptosis and their important roles in the process of LPS/CLP-induced ALI in sepsis, discusses the possible targeted drugs of the above mechanisms and their effects, describes their dilemma and prospects, and provides new perspectives for the future treatment of sepsis-induced ALI.

## 1 Introduction

Sepsis is a life-threatening organ dysfunction caused by a dysregulated host response to infection (Singer et al., 2016). The lung is one of the most vulnerable organs in sepsis, and approximately 25%–50% of patients with sepsis may develop ALI or even acute respiratory distress syndrome (ARDS) (Li W. et al., 2022). In sepsis, pathogen-associated molecular pattern (PAMP) stimulation triggers innate immune responses, whereas signal transduction by damage-associated molecular pattern (DAMP) promotes innate immune responses. It is characterized by neutrophil integration and multiple cytokines release, disrupting alveolar-capillary integrity with non-static fluid pulmonary edema and elevated permeability (Englert et al., 2019; Wu et al., 2023). The inflammatory cascade caused by excessive amplification of PAMPs and DAMPs in sepsis causes cell and tissue damage. Its clinical features include acute exacerbations, bilateral infiltrates on chest radiography, pulmonary artery wedge pressure <18 mmHg, and refractory hypoxemia (ALI, PaO2/FiO2 <300) (Bernard et al., 1994). Many studies have used LPS or CLP to establish a model of sepsis-induced ALI that models the pathological features and investigates the specific mechanisms of ALI in clinical sepsis conditions. The systemic inflammatory response triggers neutrophil and macrophage infiltration in lung tissue and release of inflammatory mediators such as tumor necrosis factor-α (TNF-α), interleukin-1β (IL-1β), and interleukin-6 (IL-6) (Xu et al., 2024). These inflammatory mediators further activate key inflammatory signaling pathways, mainly including: nuclear factor kappa-B (NF-κB), JAK2/STAT3, mitogen-activated protein kinase (MAPK), PI3K/Akt/mTOR, and Notch signaling pathways (Li W. et al., 2022). In addition, inflammatory mediators induced during sepsis can cause increased intracellular oxygen free radicals and oxides, exacerbating lung injury.

The treatment of sepsis-induced ALI/ARDS remains a key challenge for reducing sepsis-related morbidity and mortality. Most recently employed pharmacological strategies have been ineffective in reducing the morbidity and mortality associated with sepsis-induced ALI/ARDS.

Cell death supports morphogenesis during development and homeostasis after birth by removing damaged and obsolete cells. It also reduces the spread of pathogens by eliminating infected cells (Newton et al., 2024). In recent years, the types of cell death have been enriched: e.g., apoptosis, necrosis, autophagy, ferroptosis, pyroptosis. The different types of cell death could view as a single, coordinated system, in which the individual pathways are highly interconnected and can flexibly compensate for each other (Bedoui et al., 2020)^.^

Autophagy was proposed by Ashford and Porter (Ashford and Porter, 1962) in 1962 after they discovered the phenomenon of “self-eating” in cells. It is the process of engulfing one’s cytoplasmic proteins or organelles, encapsulating them into vesicles, and fusing them with lysosomes to form autophagolysosomes, which degrade the contents they contain (Zhu et al., 2022). Macroautophagy is the most common form, the process of macroautophagy includes five steps: initiation, nucleation, extension, maturation, and degradation (Cao et al., 2021; Zhu et al., 2022). Mitochondrial autophagy is a type of macroautophagy that plays important roles in early embryonic development, cell differentiation, inflammation, and apoptosis. Ubiquitination is a key modification that mediates mitochondrial autophagy, including the serine-threonine kinase PINK1 and E3 ubiquitin ligase PARKIN. BCL2L13 and FUNDC1 may also play key roles in mitochondrial autophagy (Onishi et al., 2021). Autophagy is generally regarded as a protective mechanism in cells; however, excessive levels lead to autophagic death. Reportedly, autophagy promotes cell survival or death depending on the cell type, environmental conditions, and specific stimuli (Xu et al., 2024). Previous studies have demonstrated the induction of autophagy in both septic patients and animal models with lung diseases (Newton et al., 2024). Autophagy facilitates the removal of excess inflammatory factors and suppresses lung inflammation (Racanelli et al., 2018). However, excessive autophagy exacerbates apoptosis and promotes inflammation (Chen SL. et al., 2023). In a mouse model of septic lung injury, studies have shown that autophagy levels increase during the initial phase of sepsis when the damage is localized, acting as an adaptive response to inflammation. However, as the lung injury advances to systemic inflammation, autophagy is inhibited in lung tissues. This suggests that autophagy malfunction is closely associated with the pathological development of septic lung injury (Zhao et al., 2023). However, its precise role in acute lung injury (ALI) during sepsis remains elusive.

The term ferroptosis was initially proposed by Dixon et al., in 2012 as a specific type of programmed death of iron dependence caused by lipid peroxidation with unique morphological, biochemical, and genetic features (Dixon et al., 2012). Researchers believe that ferroptotic cells often exhibit necrosis-like morphological changes, including loss of cell membrane integrity, cytoplasmic swelling, and moderate chromatin condensation. Additionally, the mitochondria of ferroptotic cells often show condensation or swelling, increased mitochondrial membrane density, reduction or disappearance of mitochondrial cristae, and outer membrane reputure (Gao et al., 2019). The important mechanisms of ferroptosis (lipid peroxidation, iron homeostasis imbalance, and antioxidant systems) have been clearly described in previous studies and will not be repeated in this paper. Excessive iron accumulation is believed to activate ferroptosis, followed by lipid peroxidation reactions that directly or indirectly lead to abnormalities in cell structure and function, ultimately leading to cell death (Chen X. et al., 2021).

Pyroptosis was first proposed by Cookson and Brennan in 2001 (Cookson and Brennan, 2001). Similar to necrotic apoptosis, both are accompanied by rupture of the plasma membrane. However, before the rupture of the plasma membrane, pyroptotic cells show less swelling and produce multiple bubble-like projections (Chen et al., 2016). The formation of cell membrane pores is accompanied by the release of cellular contents through these pores during pyroptosis. Gasdermins are a family of pore-forming effector proteins capable of causing plasma membrane rupture (Shi et al., 2017). Gasdermin D (GSDMD), a member of the gasdermin family, is a key and widely studied protein involved in pyroptosis execution. Caspases are a family of aspartate-specific cysteine proteases that have been conserved throughout evolution (Lamkanfi and Dixit, 2014). Caspases 1/3/4/5/8/11 and granzyme cleave gasdermins into two structural domains: gasdermin-N and gasdermin-C (Ding et al., 2016; Yu et al., 2021). Meanwhile, caspases directly or indirectly cleave pro-IL-1β and pro-IL-18 into their active forms (Rao et al., 2022). Consequently, the gasdermin-Ns construct gasdermin membrane pores, which release large amounts of inflammatory factors and cytoplasmic contents, resulting in cell pyroptosis (Wei et al., 2022; Dai et al., 2023). Specific mechanisms were detailed in a previous review (Yu et al., 2021). Although pyroptosis can cause some tissue damage, moderate pyroptosis eliminates intracellular pathogens during the early stages of infection (Yu et al., 2021). Conversely, excessive pyroptosis leads to an uncontrolled inflammatory response that promotes tissue and organ damage during sepsis.

**FIGURE 1:**
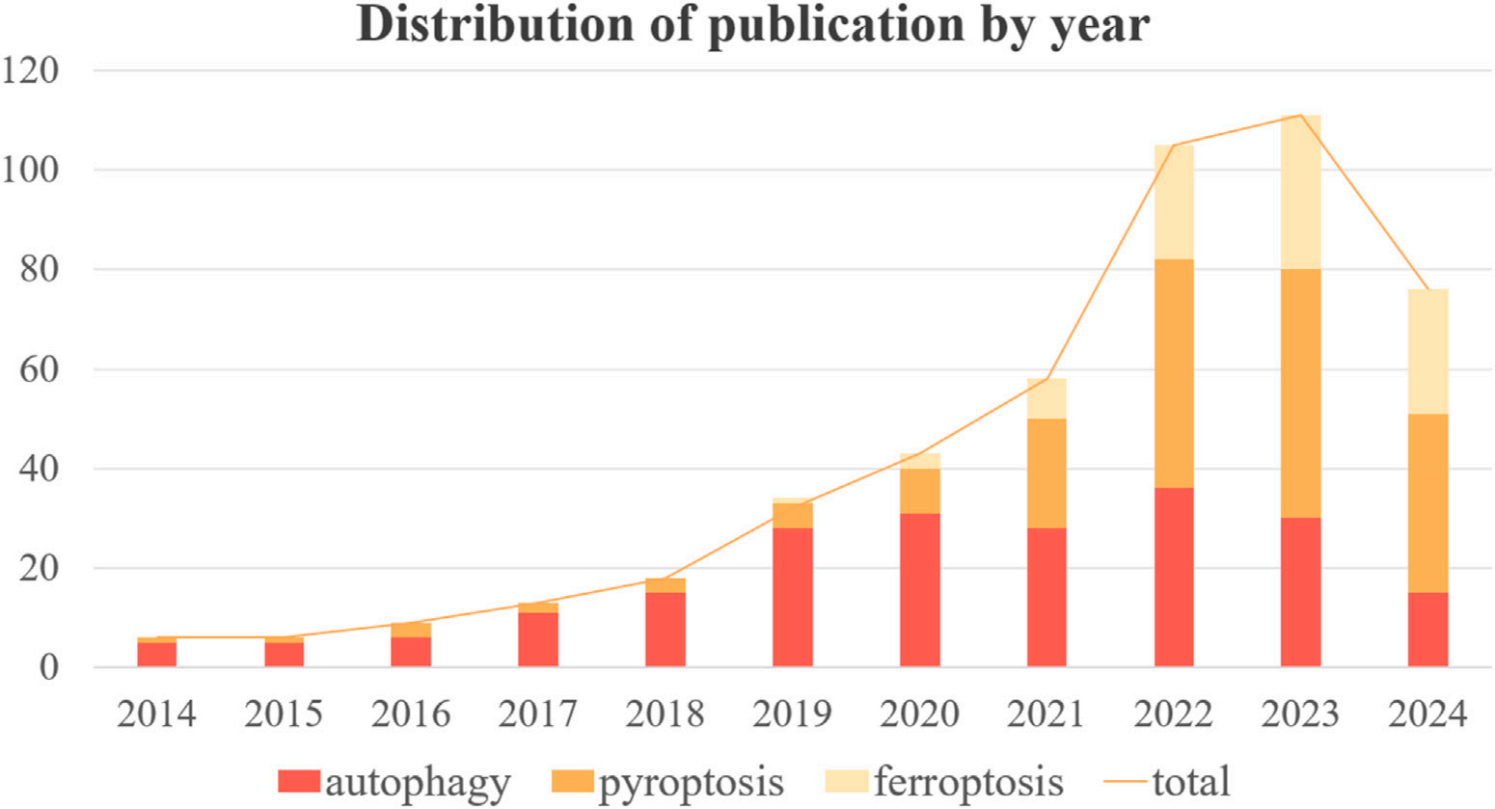
Annual trends in the number and total number of publications on autophagy, pyroptosis and ferroptosis.

**FIGURE 2:**
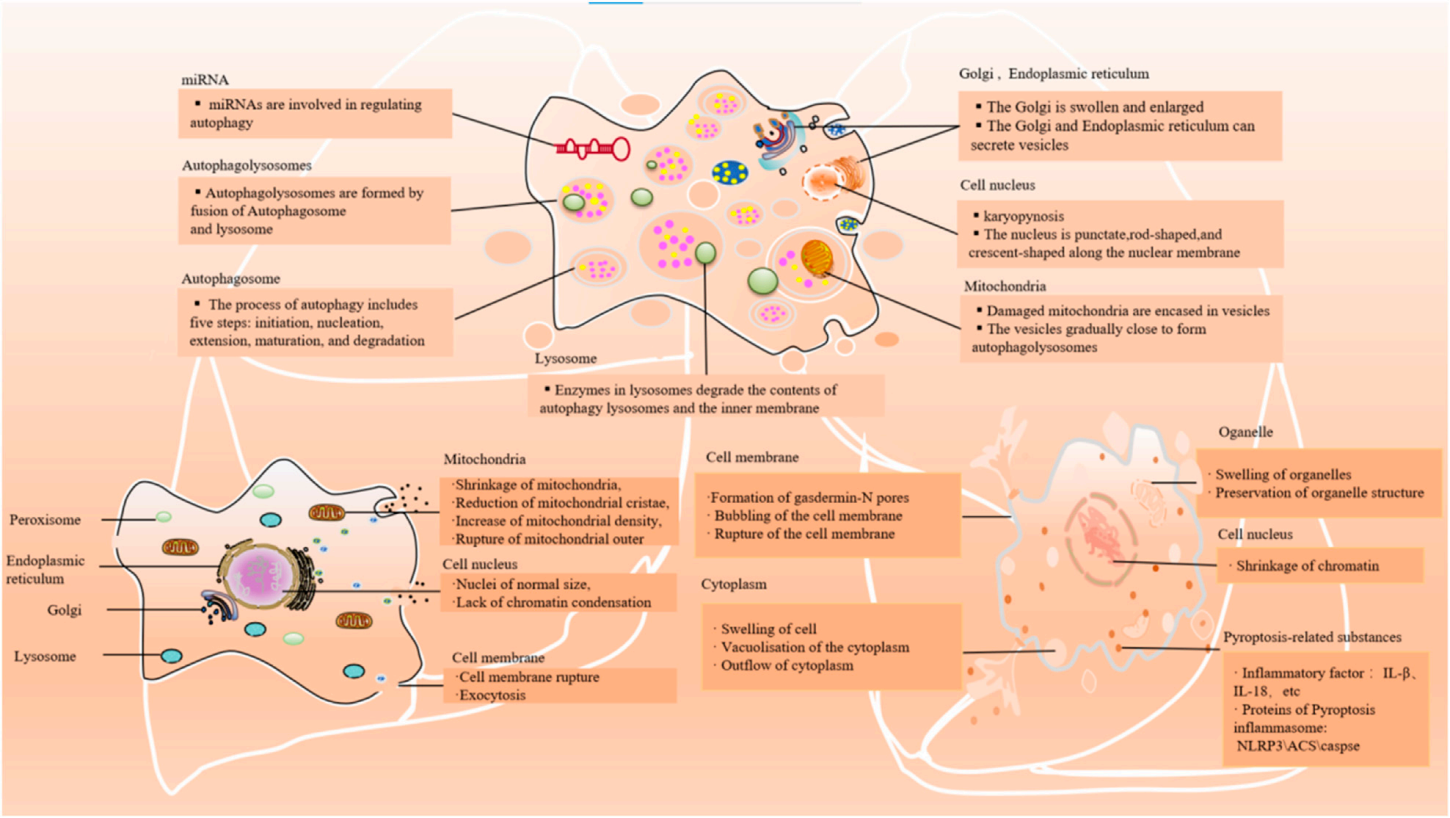
Main cellular events/features of autophagy, ferroptosis, and pyroptosis. The main morphological manifestations of autophay: expansion of Golgi apparatus and other organelles, nuclear contraction, formation of a large number of phagocytic vesicles, cell plasma membrane specialization. The main morphological manifestations of ferroptosis: shrinkage of mitochondria, reduction of mitochondrial cristae, increase of mitochondrial density, rupture of mitochondrial outer; lack of chromatin condensation and cell membrane rupture. The main morphological manifestations of pyroptosis: swelling of the cell, bubbling of the cell membrane, formation of pores in the membrane from which cytoplasmic outflow occurs; organelles are swollen but morphologically visible; and condensation of chromatin.

Autophagy, pyroptosis, and ferroptosis have emerged as prominent areas of research in the past decade. The development of ALI in sepsis is frequently associated with these three forms of programmed cell death (PCD). However, there has been a dearth of systematic investigation and analysis regarding the role played by different types of PCD in sepsis-induced ALI. In order to provide a clearer overview of the advancements made in autophagy, ferroptosis, pyroptosis, and sepsis-induced ALI, we conducted a bibliometric analysis using publications from PubMed database developed by the National Center for Biotechnology Information (NCBI) at the National Library of Medicine (NLM) spanning from 2014 to 2024. Initially, we retrieved 717 original articles; after manual screening to exclude irrelevant articles, the final count stood at 477 valid articles. The number of publications over time can serve as an indicator reflecting both the pace and trends within this field. Figure 1 illustrates the overall growth trend in annual publications along with specific counts for autophagy, ferroptosis, and pyropyrosis across each year. The total volume of publications has grown steadily from 2014–2021 with significant growth shown from 2022 to the present. As it can be noticed by the number of publications per year for each type of death, the research hotspot in the last 5 years has expanded from autophagy to pyroptosis and ferroptosis. Although the number of publications on autophagy began to decrease after 22 years, the importance of autophagy should not be overlooked. Recent studies have offered new clues to their correlation, and the study of crosstalk mechanisms between multiple death types is gaining momentum. Therefore, this review is of great clinical significance as it aims to explore the mechanisms and treatment of sepsis-induced ALI based on the regulation of autophagy, ferroptosis, and pyroptosis (Figure 2).

## 2 Possible mechanisms of programmed cell death and treatment of sepsis-induced ALI

### 2.1 The role and mechanism of autophagy in sepsis-induced ALI

#### 2.1.1 Common signaling pathways regulating autophagy in sepsis-induced ALI

##### 2.1.1.1 AMPK/mTOR signaling pathway

Autophagy is usually induced by cellular or environmental stimuli such as nutrient deprivation, metabolic and energetic stress, pathogen invasion, and oxidative stress (Shu et al., 2023). Mammalian target of rapamycin (mTOR) is a hotspot for negative autophagy regulators. mTOR is an evolutionarily conserved serine/threonine protein kinase that forms two complexes: TORC1 and TORC2 (Kim and Guan, 2015). AMP-activation protein kinase (AMPK) is an evolutionarily conserved serine/threonine protein kinase that plays key roles in energy metabolism and synthesis. It has been shown to directly promote autophagy by phosphorylating autophagy-associated proteins in the ULK1 and PIK3C3/VPS34 complex or indirectly by regulating the expression of autophagy-associated genes downstream of transcription factors [e.g., FOXO3, TFEB and BRD4] (Li and Chen, 2019). Therefore, we do not discuss these details here.

**FIGURE 3:**
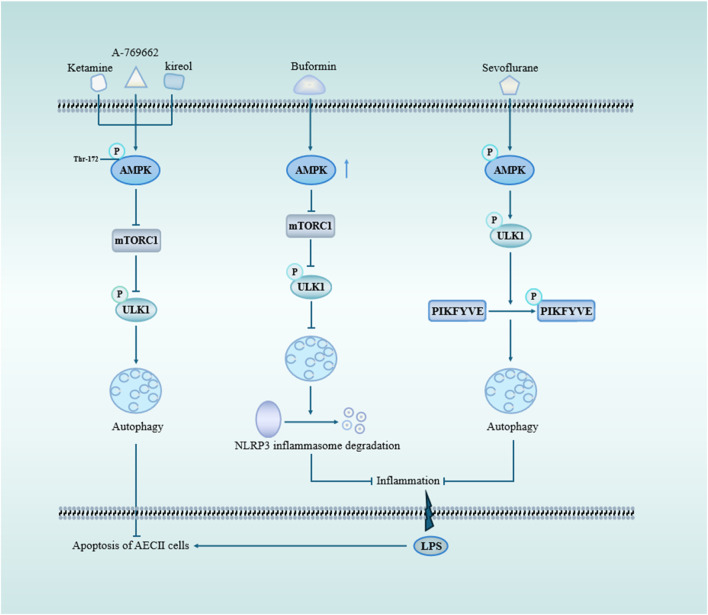
AMPK and mTOR signaling pathway through autophagy in sepsis-induced acute lung injury. mTOR serves as a pivotal negative regulator of autophagy initiation, with TORC1 being one of its crucial constituents. Upon activation of AMPK, mTOR is suppressed, leading to the phosphorylation of ULK1 and subsequent induction of autophagy. Ketamine, A-769662, and kireol can enhance autophagy while inhibiting apoptosis through the AMPK/mTOR pathway. BF promotes autophagy by upregulating AMPK expression, facilitating the degradation of NLRP3 inflammasome and mitigating LPS-induced lung inflammation. Sevoflurane-triggered activation of AMPK results in ULK1 phosphorylation, which subsequently promotes PIKFYVE phosphorylation, accelerates LC3II-to-LC3I conversion, enhances autophagosome formation, and reduces pulmonary inflammatory factors. Abbreviations: AMPK, AMP-activation protein kinase; mTORC1, mechanistic target of rapamycin complex 1; ULK1, Unc-51-like autophagy protein 1; PIKFYVE, FYVE domain-containing phosphatidylinositol 3-phosphate5-kinase.

AMPK is inhibited in inflammatory diseases (Jeon, 2016). As an important molecule involved in autophagy activation, AMPK protects against autophagy in sepsis-induced ALI (Figure 3). For example, A769662 is a member of the thienopyridone family, and Sanders et al. showed that A-769662 activates AMPK by inhibiting its dephosphorylation at Thr-172 (Sanders et al., 2007). Further studies found that A769662 ameliorated the release of inflammatory factors in sepsis-induced ALI by activating AMPK, promoting autophagy (Kitzmiller et al., 2019). The expression of AMPK was upregulated following ketamine treatment, leading to the inhibition of the mTOR pathway and subsequent promotion of autophagy, thereby enhancing apoptosis in AEC II cell (Cao et al., 2022). In the same year, Liu et al. found that buformin upregulated autophagy through an AMPK-dependent pathway, promoted NLRP3 inflammasome degradation, and inhibited NLRP3-mediated focal death in sepsis-induced ALI, which played a protective role (Liu et al., 2022). Studies have demonstrated that kirenol exerts inhibitory effects on LPS-induced inflammation through the AMPK-mTOR-ULK1 autophagy pathway (Kitzmiller et al., 2019). Additionally, there are alternative autophagy pathways independent of mTOR that can also mitigate lung injury by modulating AMPK activity. For instance, Sevoflurane attenuated inflammation, restored cellular proliferation to suppress apoptosis and sustain cell viability (Cao et al., 2022).

From these studies, various drugs and compounds can promote protective autophagy by activating AMPK, reducing inflammation, and thus protecting the ALI in sepsis. Additionally, inflammation caused by insufficient AMPK-activated autophagy may be involved in the development of sepsis-induced ALI. Meanwhile, the use of drugs to activate AMPK may be an effective way to treat sepsis-induced ALI (Table 1).

**TABLE 1:** Mechanisms of autophagy in sepsis-induced acute lung injury (ALI).

Cell death	Pathway	Compound/Target	Model	Effect	Mechanism	Ref
Autophagy	AMPK	A769662	CLP-induced septic ALI mice	Induction	A769662 inhibits mTOR signaling pathway by activating AMPK, increasing the expression of the LC3B-I and LC3B-II proteins and alleviating sepsis-induced ALI.	Kitzmiller et al. (2019)
		Buformin	LPS-induced septic ALI mice	Induction	BF inhibites NLRP3-mediated pyroptosis in sepsis-induced ALI by upregulating autophagy and Nrf2 protein levels via an AMPK-dependent pathway	Liu et al. (2022)
		Ketamine	LPS-induced septic ALI mice	Induction	AMPK expression is activated after ketamine treatment, inhibiting mTOR pathway and promoting autophagy, and alleviating the apoptosis of AEC II cells	Cao et al. (2022)
		Sevoflurane	CLP-induced septic ALI rats; MPVECs	Induction	Sevoflurane reduces inflammation, recoveres cell division to suppress cell apoptosis and maintain cell survival, and activates LPS-induced autophagic flux	Fu et al. (2022a)
	PI3K/Akt/mTOR	PICK1	CLP-induced septic ALI mice; BMDM	Induction	PICK1 has a protective effect on sepsis-induced ALI. PICK1 deficiency can inhibit the PI3K/Akt/mTOR pathway. The inhibition of Akt and mTOR pathway may affect the reformation of autolysosome disrupt the autophagic flux in PICK1^−/−^ mice, and aggravate ALI.	Mo et al. (2018), Qian et al. (2018)
		Liensinine	LPS-induced septic ALI mice; Beas-2B	Inhibition	Liensinine blocks late autophagy of LPS-induced ALI cells and attenuates LPS-induced lung injury and Inflammation	Wang et al. (2023a)
		MitoQ	CLP-induced septic ALI rats	Inhibition	MitoQ increases the expression of p-Akt, p-GSK-3β and p-mTOR, but decreases LC-3II/LC-3L levels. MitoQ inhibits autophagy to protect sepsis-induced ALI.	Li et al. (2019b)
		H2S	CLP-induced septic ALI mice; MLE-12 cells	Inhibition	Hydrogen sulfide ameliorats lipopolysaccharide-induced ALI by inhibiting autophagy via the PI3K/Akt/mTOR pathway	Zhai et al. (2015), Xu et al. (2018), Li et al. (2022b)
	PLSCR-3	Resveratrol	CLP-induced septic ALI mice; MH-S cells	Induction	Resveratrol may alleviate ALI via activating the VEGF-B signaling pathway or regulating PLSCR-3-mediated mitochondrial dysfunction and mitophagy	Yang et al. (2018), Wang et al. (2021)
	PINK1 /Parkin	Hydrogen	CLP-induced septic ALI mice; RAW 264.7 cells	Induction	Hydrogen inhibits ALI in CLP mice via activation of PINK1-mediated mitophagy	Chen et al. (2021b)
	PINK1 /Parkin	Resveratrol	LPS-induced septic ALI mice; AM cells	Induction	Resveratrol alleviates mitochondrial damage by promoting mitophagy, subsequently inhibiting NLRP3 inflammasome activation and reducing the release of pro-inflammatory mediators. Resveratrol-induced mitophagy is associated with the Pink/Parkin signaling pathway and found that mitophagy is halted in the absence of Pink1	Wu et al. (2024)
	TRAF3/ULK1/NLRP3	—	LPS-induced septic ALI mice; THP-1 cells and mouse bone marrow macrophages	Inhibition	This research highlights the TRAF3-ULK1-NLRP3 regulatory axis as a pivotal pathway in ALI development and suggests that targeting this axis could be an effective therapeutic strategy for ALI treatment	Jiang et al. (2024)

##### 2.1.1.2 PI3K/Akt/mTOR signaling pathway

In sepsis, bacteria or viruses often activate PI3K/Akt, promoting the infiltration of inflammatory cells in the lung by regulating endothelial cell injury (Margaria et al., 2022). Phosphatidylinositol 3-kinase (PI3K) is an intracellular phosphatidylinositol kinase with phosphatidylinositol and serine/threonine kinase activities. PI3K is divided into three classes, and I PI3K is the most widely studied. PI3K activation catalyzes the production of PIP3 (Geering et al., 2007), acting as a secondary messenger. PIP3 can recruit PDK1 and Akt proteins to the plasma membrane through its pleckstrin homology structural domain, causing PDK1 to phosphorylate threonine at position 308 of AKT, leading to AKT activation (Alessi et al., 1997; Stokoe et al., 1997). AKT is a class of serine/threonine kinases in the AGC family that plays a key role as a proto-oncogene in various cellular regulations (Revathidevi and Munirajan, 2019). PI3K/Akt signaling pathway is key in mediating cellular responses to inflammatory reactions (Tian et al., 2023). As a negative autophagy regulator, AKT indirectly enhances mTOR activation by Rheb through inhibition of TSC2 (Yang et al., 2006; Huang and Manning, 2009). mTORC1 is a substrate of AKT. Activated TORC1 inhibits autophagy-related proteins, such as the downstream substrate ULK1, by phosphorylating it to inhibit autophagy (Wang et al., 2012; Noda, 2017). P62 is an important bridging protein that binds to substrates to facilitate their entry into autophagosomes and plays an important role in autophagic lysosomal degradation (Liu et al., 2016). LC3 II/I and P62 are widely used to measure autophagy fluxes (Klionsky et al., 2008).

Autophagy regulation via the PI3K/Akt/mTOR si PI3K/Akt/mTOR gnaling pathway is beneficial in sepsis-induced ALI. PICK1 is a peripheral membrane protein conserved from Cryptobacterium hydradii to humans that is expressed in many tissues and plays important roles in cellular localization, lipid regulation, and protein trafficking (Xu and Xia, 2006). A previous study reported that PICK1 knockout mice receiving CLP exacerbated ALI (Qian et al., 2018). Another study revealed that PICK1 knockout mice exhibited increased levels of phosphorylated p-Akt and p-mTOR, indicating the suppression of early-stage autophagy. Moreover, deficiency in PICK1 disrupts lysosome structure and leads to dysfunction in late-stage autophagy as well as accumulation of autophagosomes, thereby exacerbating sepsis-induced ALI (Xu and Xia, 2006). This study suggests that PICK1 regulates the generation of autophagosomes by inhibiting the PI3K/Akt/mTOR signaling pathway, thus exerting a protective effect against sepsis-induced lung injury. Therefore, autophagy dysfunction caused by insufficient PICK1 protein expression may be involved in sepsis-induced ALI.

Previous studies have shown that excessive autophagy activation of alveolar type II epithelial cells was a key feature of aggravated ALI (Song L. et al., 2017). Another study demonstrated the beneficial effects of inhibiting late-stage autophagy. Through the PI3K/Akt/mTOR pathway, liensinine blocks the binding of autophagosomes to lysosomes, induces the accumulation of autophagosomes. This change may be responsible for the increased viability of alveolar epithelial cells and the decreased release of inflammatory factors (Wang C. et al., 2023).

In addition to the inhibition of advanced autophagy by the above-mentioned liensinine to reduce lung injury, Hydrogen is currently a common research object in regulating autophagy to reduce lung injury through the PI3K/Akt/mTOR signaling pathway. Moreover, as early as 2015, treatment with hydrogen-rich saline could effectively improve inflammation in sepsis-induced ALI (Zhai et al., 2015). In 2018, it was discovered that hydrogen sulfide can activate the PI3K/Akt/mTOR pathway to suppress excessive autophagy activation, thereby mitigating LPS-induced reduction in cell viability and LDH release, ultimately ameliorating lung injury (Xu et al., 2018). Further, GYY4137 (a novel H2S donor) inhibited the activation of autophagy and attenuated ferroptosis, which reduced lung injury (Li J. et al., 2022).

#### 2.1.2 miRNAs are involved in sepsis-induced ALI by regulating autophagy

According to genome-wide expression analyses, approximately 80% of genetic elements are aberrantly expressed in patients with sepsis. Non-coding RNAs, including micro RNAs (miRNAs), long non-coding RNAs, and cyclic RNAs, are key regulators of sepsis pathogenesis (Zhang et al., 2017). miRNAs, approximately 21 nucleotides in length, are a large family of post-transcriptional regulators of gene expression that control many developmental and cellular processes in eukaryotes (Krol et al., 2010). New therapeutic approaches focusing on miRNA intervention have received considerable attention (Chen et al., 2020). The role of miRNAs in regulating autophagy and sepsis-induced ALI is complex. They can either play a protective role or aggravate disease progression. These effects may be related to various factors, such as cell type and drug dosage, the mechanisms of which remain unclear. Since autophagy regulated by miRNAs plays an important role in sepsis-induced ALI, it is important to study the mechanism of miRNA-mediated autophagy regulation for treating sepsis-induced ALI. This study reviews the mechanisms of autophagy regulation from the protective and harmful aspects of miRNAs to provide new ideas for treating diseases.

##### 2.1.2.1 Protection of miRNA overexpression

Different miRNAs may have different effects on autophagy in the same type of cell. Beclin 1 and β-activated kinase 1-binding proteins 2 (TAB2) are important autophagy mediators. Overexpression of TAB2 C-terminal domains and Beclin 1 CCD structural domain competitively destroyed endogenous Beclin 1 and inhibited autophagy, whereas TAB2’s absence triggered autophagy (Criollo et al., 2011). IL-1β and TNF-α directly cause lung injury in sepsis-induced ALI by damaging the capillary endothelium and alveolar epithelium. Inactivation of Caspase-1 reduces IL-1 synthesis. miR-155 induces autophagy by inhibiting TAB2, resulting in reduced TNF-α and IL-1 levels, as well as decreased Caspase-1 expression in lung macrophage cells, thereby ameliorating lung injury (Liu F. et al., 2017). Another study found that, BMSC-derived exosomes can reduce lung tissue damage by transferring miR-384-5p into alveolar macrophages. miR-384-5p Directly binds to Beclin-1, inhibiting LPS-induced macrophage autophagy. This helps in attenuating extensive leukocyte infiltrates, interstitial and alveolar edema, and lung hemorrhage (Liu X. et al., 2021). The two studies appear to have conflicting findings. Although they both observed an increase in macrophage autophagy, the increase in miR-155 attenuated lung injury while the decrease in miR-384-5p aggravated it. Since cellular injury involves multiple mechanisms and the human body is highly complex, further in-depth research is necessary to understand how to effectively regulate autophagy in order to alleviate sepsis-induced ALI.

**FIGURE 4:**
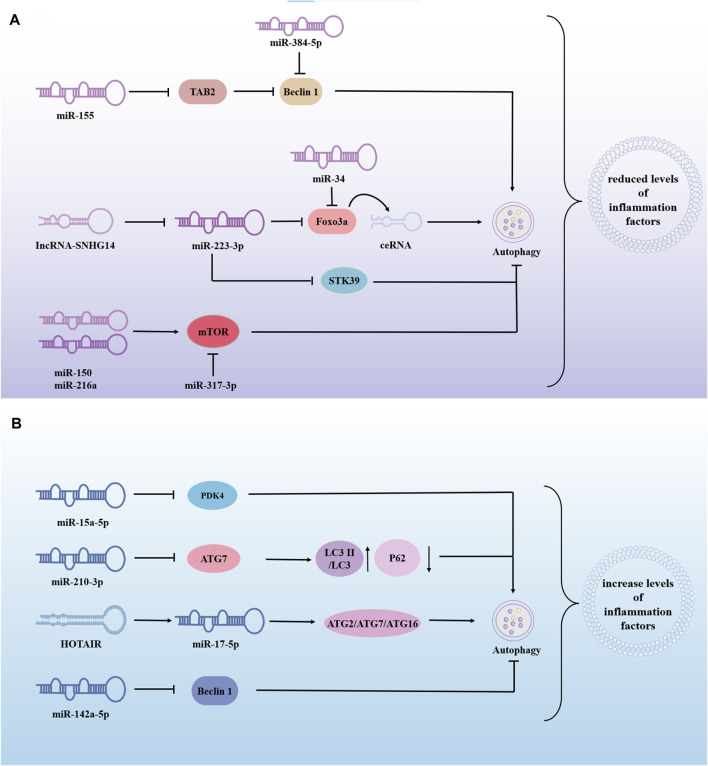
The role of miRNA-modified autophagy in sepsis-induced acute lung injury (ALI). miRNAs are involved in sepsis-induced ALI by regulating autophagy, to inhibite or promote disease progression. **(A)** Overexpression of miRNA alleviates lung injury by promoting or inhibiting autophagy. **(B)** Overexpression of miRNA exacerbates sepsis-induced ALI by promoting or inhibiting autophagy. → indicates a promoting effect and ⊥ indicates an inhibitory effect. Abbreviations: TAB2, β-activated kinase 1-binding proteins 2; STK39, Recombinant Serine/Threonine Kinase 39; PDK4, pyruvate dehydrogenase kinase isozyme 4; ATG7, autophagy associated genes 7; LC3II, Human microtubule-associated protein light chain 3II.

The same miRNA can either promote or inhibit autophagy, thereby exerting varying effects on the progression of sepsis-induced ALI. This dual role may be influenced by factors such as cell type and disease progression, requiring further investigation. For example, MiR-223-3p-loaded exosomes from the bronchoalveolar lavage fluid promote alveolar macrophage autophagy, increase cell viability and reduces the release of inflammation, thereby attenuating ALI by inhibiting the expression of Recombinant Serine/Threonine Kinase 39(STK39) (He N. et al., 2022). Alveolar type II epithelial cells help maintain alveoli structure and function and assist in repair after injury. Research shows that LPS-treated alveolar type II epithelial cells had decreased miR-223-3p and increased Foxo3a, affecting autophagy. Downregulation of lncRNA-SNHG14 or upregulation of miR-223-3p can reduce IL-6, IL-1β, and TNF-α levels by inhibiting autophagy (Hong et al., 2021). In addition, upstream regulation of FOXO3 is not limited to miR-223-3p. miR-34a might suppress the excessive autophagic activity in AT-II cells by targeting FOXO3 to reduce the damage caused by LPS-induced ALI (Song L. et al., 2017). Similarly, the overexpression of miR-216a and miR-150 inhibited autophagy and alleviated LPS-induced ALI (Li P. et al., 2019; Kong et al., 2020). miR-377-3p released by hucMSC exosomes ameliorates Lipopolysaccharide-induced ALI by targeting RPTOR to induce autophagy *in vivo* and *in vitro* (Wei et al., 2020). From these studies, it can be concluded that the overexpression of miRNAs can reduce LPS-induced ALI by promoting or inhibiting autophagy (Figure 4A), and most of the miRNAs in these studies had low or lower expressions than normal people in LPS-treated cells or patients with sepsis. This suggests that these miRNAs participate in the progression of sepsis-induced ALI by regulating autophagy and promoting protective miRNAs to provide a new direction for disease treatment.

##### 2.1.2.2 Harmfulness of miRNA overexpression

miR-210-3p is highly enriched during sepsis and is released into the bloodstream to promote leukocyte migration. miR-210-3p overexpression decreases autophagy associated genes 7 (ATG 7) and LC3II/LC3I expression and increases P62 expression. Treatment of septic mice with adenovirus-anti-miR-7-3p improved the impaired autophagosome formation caused by miR-210-3p overexpression (Li G. et al., 2021). HOX transcript antisense intergenic RNA(HOTAIR) regulated apoptosis, cell cycle, proliferation and autophagy through the miR-17-5p/ATG2/ATG7/ATG16 axis, thereby driving LPS-induced ALI. HOTAIR knockdown (si-HOTAIR) suppressed protein expression of the autophagy markers light chain 3B and Beclin-1, and alleviated LPS-induced lung injury *in vivo* (Li Y. et al., 2021). MSCs may alleviate LPS-ALI through downregulating miR-142a-5p, which allows PECs to increase Beclin-1-mediated cell autophagy (Zhou and You, 2016). Negatively mediated by Protein kinase C-alpha (PPKCA), miR-15a-5p was highly expressed in LPS/IFN-γ-treated macrophages. miR-15a-5p overexpression inhibits mitochondrial autophagy by directly targeting PDK4. PPKCA overexpression can negatively regulate the miR-15a-5p/PDK4 pathway to promote mitochondrial autophagy and alleviate sepsis-induced ALI (Zhu et al., 2023). The above studies show that miRNAs can also promote sepsis-induced ALI in some cases (Figure 4B). Inhibition or downregulation of the expression of harmful miRNAs can significantly reduce lung injury and provide new targets for treating sepsis-induced ALI. Therefore, more effective drugs and targets need to be identified. In addition, ATG may be the key center of miRNA regulation of autophagy, thereby aggravating or alleviating lung injury in sepsis.

#### 2.1.3 mtDNA is involved in sepsis-induced ALI by regulating autophagy

Mitochondria are central to the cellular energy supply. The regulation of mitochondrial autophagy plays an important role in the progression of sepsis-induced ALI (Table 1). RUNX1-dependent activation of mitophagy in AT2 protects the lungs from LPS injury. This also supports the conclusion that dysregulation of RUNX1-dependent mitophagy in AT2 cells contributes to the pathogenesis of ARDS (Tang et al., 2023). In a CLP-induced mouse model, resveratrol alleviated ALI by modulating PLSCR-3-mediated mitochondrial dysfunction and autophagy (Wang et al., 2021). The PINK1/Parkin pathway, a key regulator of mitochondrial autophagy, is activated to promote mitochondrial autophagy in response to H, which, in turn, inhibits ALI in CLP (Chen H. et al., 2021). Resveratrol has been found to mitigate mitochondrial damage by promoting mitophagy, thereby facilitating the timely elimination of damaged mitochondria and inhibiting inflammasome activation and the release of proinflammatory factors. This mechanism aids in maintaining intracellular homeostasis and alleviating inflammatory cell infiltration, hemorrhage, and alveolar septal thickening (Chen H. et al., 2021). Another experiment validated the interaction between autophagy and NLRP3. Upregulation of ULK1 was shown to contribute to enhanced LPS-induced autophagy. NLRP3 was identified as a pivotal pyroptosis gene implicated in ALI development. The TRAF3 protein was discovered to inhibit ULK1 through ubiquitination, leading to increased activation of the NLRP3 inflammasome. Their activation can trigger initiation, amplification, and injury associated with inflammation in ALI (Taanman, 1999).

**FIGURE 5:**
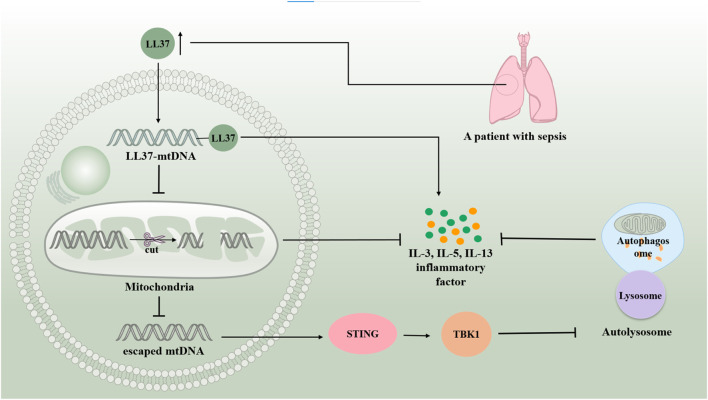
mtDNA is involved in sepsis-induced acute lung injury by regulating autophagy. mtDNA can escape autophagy and cause inflammation. Serum human antimicrobial peptide LL-37 levels are higher in patients with severe sepsis than in those with mild sepsis. LL37-mtDNA complex avoids the degradation of mtDNA by DNase II, allowing it to escape autophagy. The excess of mtDNA activates the STING/TBKI pathway, which results in abnormal autophagy and exacerbating sepsis-induced acute lung injury.

Human mtDNA is a 16,569 bp double-stranded circular molecule containing 37 genes encoding two rRNAs, 22 tRNAs, and 13 polypeptides. mtDNA-encoded polypeptides are subunits of the enzyme complex of the oxidative phosphorylation system (Taanman, 1999). Damage to mtDNA and loss of mitochondrial genome integrity play roles in the development of severe early onset and chronic age-related diseases (Sharma and Sampath, 2019) (Figure 5.). It has been found that mtDNA can escape from autophagy and cause inflammation. mtDNA, an inflammatory factor, cannot escape autophagy or DNase II degradation. However, atherosclerotic plasma and plaques have elevated levels of the LL37-mtDNA complex, which prevents the degradation of mtDNA by DNase II and allows it to escape autophagy (Zhang et al., 2015). Reportedly, serum human antimicrobial peptide LL-37 levels were higher in patients with severe sepsis than in those with mild sepsis. Exogenous delivery of LL-37-mtDNA complex significantly exacerbated lung inflammation. Anti-cramp-mtDNA antibodies attenuate the hyperinflammatory response in LPS-induced acute lung injury. Treatment with LL-37 may effectively reduce mtDNA degradation to inhibit mitochondrial autophagy, thereby exacerbating septic ALI (Zuo et al., 2019). This reveals a novel mechanism of autophagy in septic lung injury. Additionally, infection and stress promote the release of mtDNA from injured cells into circulation. Excess mtDNA activates the STING pathway, which induces lysosomal acidification disorders in an interferon-dependent manner via the TBK1 signaling pathway. This results in abnormal autophagy and exacerbation of sepsis. Induced autophagy and lack of STING could attenuate lung injury, revealing the involvement of autophagy disorder in the sepsis-ALI Pathway (Liu Q. et al., 2021). Thus, it is clear that inhibition of mitochondrial autophagy generally aggravates ALI in sepsis. The autophagic escape observed in patients with severe sepsis may be the key to aggravation.

Based on these studies, the mechanisms associated with autophagy and ALI in sepsis are complex. In different cases, insufficient or excessive autophagy can cause lung injury. Promoting or inhibiting autophagy may serve as mechanisms to protect against lung injury in sepsis. Autophagy, as a conservative mechanism for maintaining cellular homeostasis, is responsible for degrading misfolded proteins, damaged organelles, and intracellular pathogens (Glick et al., 2010). We propose that activation of autophagy at an early stage could restore cell vitality by phagocytosing damaged organelles and inflammatory factors, thereby reducing lung injury. However, during the later stages of the disease, autophagy alone is insufficient to counteract the extensive inflammatory damage; excessive activation of autophagy in alveolar epithelium accelerates cell death (Song L. et al., 2017). At this stage, autophagy may inadvertently degrade normal proteins and organelles, exacerbating cell damage and hastening the progression of lung injury in sepsis. Various signaling pathways that regulate autophagy, such as miRNAs and mtDNAs, are involved in disease progression. Therapies targeting each molecule or gene open up a new market for treating lung injury in sepsis.

### 2.2 Role and mechanisms of ferroptosis in sepsis-induced acute lung injury

#### 2.2.1 P53 signaling pathway

Tumor protein p53 (P53) is a human tumor suppressor gene that mediates cell cycle arrest, apoptosis, and metabolic changes (Riley et al., 2008). It involves many other physiological and pathological processes (e.g., immune response, tissue ischemia/reperfusion, neurodegeneration). In 2015, Le Jiang et al. linked P53 to ferroptosis for the first time, claiming that P53 and mutant P53 were involved in regulating ferroptosis (Jiang et al., 2015a). Studies since then have demonstrated the dual role of P53 in regulating ferroptosis. It can enhance ferroptosis by inhibiting solute carrier family 7 member 11 (SLC7A11) expression or promoting SAT1 and GLS2 expression. In contrast, P53 can inhibit ferroptosis by suppressing DPP4 activity or inducing CDKN1A/p21 expression (Jiang et al., 2015a; Jiang et al., 2015b; Kang et al., 2019). SLC7A11, an important component of the Xc-GSH-GPX4 antioxidant pathway, promotes the exchange of cysteine and glutamate in the plasma membrane. In this antioxidant pathway, the system Xc, which helps glutathione peroxidase 4 (GPX4) reduce peroxides, counteracts ferroptosis by transporting cysteine to synthesize glutathione (Dixon et al., 2012; Dixon et al., 2014).

Meteorin-like/Meteorin-β (Metrnβ), also known as interleukin (IL)-41, is a newly discovered secreted protein. Researchers have experimentally demonstrated that it protects against LPS-induced ALI by inhibiting ferroptosis by activating of the SIRT1-P53-SLC7A11 signaling pathway (Chen Z. et al., 2023). A study by Youjing Yang et al. claimed that STAT6 deficiency in the lung epithelium promotes ferroptosis and exacerbates lung injury: more inflammatory cell infiltration and thickened alveolar septum. They demonstrated that signal transducer and activator of transcription 6 (STAT6) attenuates ALI by inhibiting ferroptosis while increasing the cellular antioxidant capacity, which inhibits the expression of SLC7A11, the binding of P53 and CREB-binding protein, and decreases the acetylaEEEtion of P53 by competitively binding to CREB-binding protein (Yang et al., 2022). It was claimed that MLK3 knockdown attenuated LPS-induced cellular injury by blocking the p53 pathway (Chen et al., 2022). Notably, P53 has a complex and extensive control of ferroptosis and is important for treating the disease. Currently, research on the P53 signaling pathway and ferroptosis is more related to cancer and less on sepsis-induced ALI; further exploration is needed to understand how to utilize this regulatory mechanism to inaugurate an emerging therapeutic area for sepsis-induced ALI in the future.

#### 2.2.2 ACSL4 signaling pathway

Acyl-CoA synthetase long-chain family member 4 (ACSL4) is mainly located in the endoplasmic reticulum, mitochondria, plasma membrane, and peroxisomes, abundant in adrenal glands, ovaries, testes, and brain tissues (Quan et al., 2021). ACSL4 affects ferroptosis by altering cellular lipid composition, catalyzed by ACSL4 and lysophosphatidylcholine acyltransferase 3. Polyunsaturated fatty acid (PUFA), such as arachidonic acid and epinephrine, synthesize lipids sequentially and are inserted into membrane phospholipids to form the PUFA-PL complex (Yuan et al., 2016; Doll et al., 2017). Subsequently, they are oxidized to lipid hydroperoxides in the presence of peroxidases, activating ferroptosis (Kagan et al., 2017). Activation of iron-dependent lipid peroxidation produces lipid peroxides (malondialdehyde and 4-hydroxynonenal), which can lead to the formation of holes in the lipid bilayer, ultimately leading to cell death (Tang and Kroemer, 2020).

Reportedly, LPS stimulation upregulates ACSL4 expression, which is blocked by uridine supplementation. Uridine treatment attenuated sepsis-induced ALI pathological changes, as manifested by pulmonary hemorrhage, interstitial edema and thickening of the alveolar wall in HE staining. This maybe because uridine promotes excess cellular energy and lipolysis and inhibits lipid synthesis, indicating that uridine exerts a protective effect against ferroptosis through the ACSL4 signaling pathway (Lai et al., 2023). CircEXOC5 enhances the stability of its target gene ACSL4 by binding to the RNA-binding protein PTBP4 and up-regulating its expression, thereby promoting ferroptosis and exacerbating sepsis-induced ALI. The results of the study showed that the CLP group showed vascular congestion, hemorrhage, alveolar sac collapse, alveolar wall and alveolar septum thickening (Wang W. et al., 2022). The knockdown of SHP2 downregulated ACSL4 expression to attenuate ferroptosis in LPS-induced ALI 2 (Li et al., 2023). These studies clearly show that ferroptosis can be controlled through the ACSL4 signaling pathway for cell protection; however, more of these mechanisms need further investigation to provide ideas for the clinical treatment of sepsis-induced ALI.

#### 2.2.3 Nrf2 signaling pathway

Nuclear factor erythroid 2-related factor 2 (Nrf2) is a transcription factor with seven functional structural domains responsible for maintaining the stability of Nrf2 and regulating its transcriptional activity (Bellezza et al., 2018). Nrf2 is at low levels under normal conditions. Once oxidative stress occurs, it rapidly moves from the cytoplasm to the nucleus, where it binds to antioxidant/electrophilic response elements (AREs/EpREs) to regulate the redox state of cells (Itoh et al., 1997). As an essential cofactor, iron participates in various physiological and biochemical reactions, simultaneously iron may increase oxidative damage by directly generating excess reactive oxygen species (ROS) through the Fenton reaction. Therefore, maintaining iron homeostasis is fundamental for various physiological and biochemical reactions, and when iron overload is predisposed to ferroptosis (Dixon et al., 2012; Doll and Conrad, 2017). Nrf2 maintains iron homeostasis by participating in several reactions that regulate haem synthesis, hemoglobin catabolism, iron storage, and iron transfer. Therefore, Nrf2 is very important in regulating ferroptosis (Kerins and Ooi, 2018).

**FIGURE 6:**
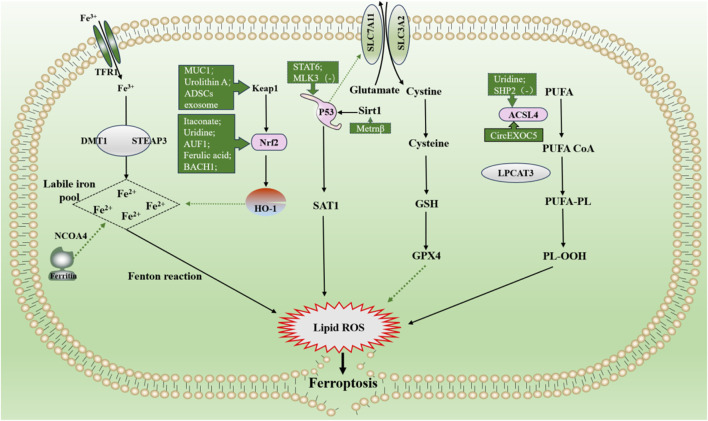
Main mechanism and regulation of ferroptosis in sepsis-induced acute lung injury. It can be roughly divided into three categories. I: iron metabolism pathways. The extracellular ferric iron is oxidized to ferrous iron and transported into the cell, and the excess iron ions undergo the Fenton reaction to generate a large amount of reactive oxygen species (ROS). II: lipid peroxidation. PUFA generate a large amount of lipid peroxides under the action of a series of enzymes. Accumulation of lipid ROS leads to ferroptosis. III: antioxidant system. Cystine is transported into cells by the system Xc and then reduced to cysteine, which together with glutamate and glycine synthesize glutathione, promote the synthesis of GPX4, remove intracellular lipid ROS, and inhibit ferroptosis. The solid line indicates the promotion and the dotted line indicates the inhibition. Dotted arrows show inhibition, solid arrows show promotion. Abbreviations: GSH, glutathione; GPX4, glutathione peroxidase 4; NOCOA4, nuclear receptor coactivator 4; SAT1, spermidine/spermine N1-acetyltransferase 1; PUFA, polyunsaturated fatty acids; PL-OOH, phospholipid hydroperoxides.

Itaconate is produced by diverting aconitate away from the tricarboxylic acid cycle during macrophage activation. 4-octyl itaconate (4-OI), a cell-permeable derivative of endogenous itaconate, has the potential to activate Nrf2 pathways. HE R et al. found that pre-treatment of 4-OI significantly attenuated LPS-induced ALI, as reflected by pulmonary hemorrhage, interstitial edema, thickening of the alveolar wall, and tissue damage. Their findings suggest that itaconate promoted the transcription of target genes (GPX4, GCLM, and SLC7A11) and inhibited macrophage ferroptosis in an Nrf2-dependent manner (He R. et al., 2022). Moreover, uridine promotes the expression of Nrf2 in cells, animals, and patients with sepsis, leading to an increase in Nrf2-dependent antioxidant targeting genes (SLC7A11, GPX4, and HO1), indicating that it activates the antioxidant system and inhibits ferroptosis (Lai et al., 2023). Although studies have shown that Nrf2 inhibits ferroptosis by up regulating GPX4, there are differences in the specific, ways in which AUF1 upregulates Nrf2 through mRNA levels, and MUC1 enhances the inhibitory effect of vitamin E on Keap1 and stimulates the phosphorylation level of GSK3β to promote Nrf2 entry into the nucleus (Wang YM. et al., 2022; Wang Y. et al., 2022). ADSCs exosomes inhibited sepsis-induced ALI caused by ferroptosis in PMVECs by inhibiting Keap1 to upregulate GPX4 expression and facilitate Nrf2 expression and translocation into the nucleus (Shen et al., 2023). The specific pathological manifestations of ALI: alveolar capillary swelling and congestion, alveolar cavity bleeding, inflammatory cell infiltration, have been improved (Shen et al., 2023). Prior research has revealed that Urolithin A (UA) has different pharmacological properties, including antioxidation, anti-inflammation, neuroprotection, and improved muscle function. The results of the study by LOU L et al., in 2023 show that UA inhibits LPS-mediated inflammation and ferroptosis through activation of the Keap1-Nrf2/HO-1 pathway and is effective in the treatment of ALI (Lou et al., 2023). Avic acid inhibited ferroptosis and attenuated alveolar epithelial barrier dysfunction in sepsis-induced ALI by activating the Nrf2/HO-1 pathway (Tang et al., 2022). BACH1 is a novel LPS-induced injury regulator that modulates inflammatory responses, oxidative stress, and ferroptosis by activating Nrf2/HO-1 signaling, suggesting that BACH1 may be a promising therapeutic candidate for ALI treatment (Wang RX. et al., 2023). In summary, Nrf2 inhibited ferroptosis by inhibiting peroxidation and maintaining iron homeostasis, alleviating sepsis-induced ALI. Consequently, Nrf2 may be a future target for treating sepsis-induced ALI, which requires further investigation (Figure 6).

#### 2.2.4 Other signaling pathways

In addition to the mechanisms described above, other signaling pathways can regulate ferroptosis to achieve a protective effect against sepsis-induced ALI. For example, nuclear receptor coactivator 4 (NCOA4) is a selective cargo receptor for ferritin. NCOA4-dependent autophagy, also known as ferritin phagocytosis, causes an increase in intracellular iron levels and the Fenton reaction, which promotes the onset of ferroptosis (Hou et al., 2016). YAP1 disrupted the NCOA4-FTH1 response and inhibited NCOA4-mediated ferritin phagocytosis, preventing ferroptosis and subsequent mitochondrial ROS-related dysfunction in sepsis-induced ALI (Zhang et al., 2022). Protectin conjugates in tissue regeneration 1 (PCTR1) in tissue regeneration is an endogenous lipid mediator that inhibits LPS-induced ferroptosis through the ALX/PKA/CREB signaling pathway (Lv et al., 2023). Additionally, pretreatment with rmMANF attenuates sepsis-associated lung injury by inhibiting the GRP78-dependent PERK/ATF4 pathway and ferroptosis in mice (Zeng et al., 2023). STING promotes ferroptosis through STAT3 signaling, promoting LPS-induced ALI (Gu et al., 2024). A large number of extracellular neutrophil extracellular traps (NETs) are produced during the development in sepsis patients and experimental models. Excessive NETs can damage the endothelial glycocalyx, leading to disruption of the integrity of the SDC-1/HS/HGF complex, thereby obstructing the downstream cMET signaling pathway conduction and inducing endothelial ferroptosis, which further exacerbates tissue injury (Fei et al., 2024). Therefore, these approaches could provide new ideas and targets for the treatment of sepsis-induced ALI(Table 2).

**TABLE 2:** Mechanisms of ferroptosis in sepsis-induced acute lung injury (ALI).

Cell death	Pathway	Compound/Target	Model	Effect	Mechanism	Ref
Ferroptosis	P53	Meteorin-like/Meteorin-β (Metrnβ)	LPS induced-septic ALI mice; MLE-12 cells	Inhibition	Metrnβ regulates ferroptosis in ALI by modulating the SIRT1-P53- SLC7A11 pathway lung tissue	Chen et al. (2023b)
		STAT6	LPS induced-septic ALI mice; HBE cells/THP-1 cells	Inhibition	STAT6 negatively regulated ferroptosis via regulating the P53/SLC7A11 pathway	Yang et al. (2022)
		MLK3	MLE-12 cells	Inhibition	Silence of MLK3 alleviated LPS-induced lung epithelial cell injury by inhibiting p53-mediated ferroptosis	Chen et al. (2022)
	ACSL4	CircEXOC5	CLP induced-septic ALI mice; MPVECs	Induction	CircEXOC5 can enhance the stability of the target gene ACSL4 by binding to the RNA binding protein PTBP1 and upregulate its expression, thereby promoting ferroptosis and exacerbating sepsis-induced ALI	Wang et al. (2022a)
		Uridine	LPS induced-septic ALI mice; THP-1/A549/HUVEC cells	Inhibition	Uridine supplementation prevents upregulated ACSL4 expression by promoting cell energy excess, boosting lipolysis, and inhibiting lipid synthesis	Lai et al. (2023)
		SHP2	LPS induced-septic ALI mice; MLE-12 cells	inhibition	Knockdown of SHP2 reduced the ACSL4 expression, subsequently decreasing the accumulation of ROS and the susceptibility to ferroptosis, thereby leading to the alleviation of ferroptosis and LPS-induced ALI	Li et al. (2023)
	Nrf2	Itaconate	LPS induced-septic ALI mice; THP-1 cells	Inhibition	The function of itaconate 4-OI to inhibit macrophage ferroptosis was dependent on the blockage of the degradation of Nrf2, the resultant increase of Nrf2 promoted the transcription of target genes, including GPX4, GCLM, SLC7A11	He et al. (2022b)
		Uridine	LPS induced-septic ALI mice; THP-1/A549/HUVEC cells	Inhibition	Uridine supplementation prevents upregulated ACSL4 expression by promoting cell energy excess, boosting lipolysis, and inhibiting lipid synthesis	Lai et al. (2023)
		AUF1	CLP induced-septic ALI mice; AECS cells	Inhibition	AUF1 stabilized the stability of Nrf2 mRNA and at the same time promoted the degradation of ATF3 mRNA, and both were functionally important for resistance to ferroptosis	Wang et al. (2022c)
		MUC1	CLP induced-septic ALI mice; MLE-12 cells	Inhibition	MUC1 can inhibit Keap1, increase the phosphorylation level of GSK3β, and promote Nrf2 entry into the nucleus, thus improving the expression level of GPX4, sensitizing vitamin E, inhibiting ferroptosis, and alleviating acute lung injury in sepsis	Wang et al. (2022b)
		ADSCs exosome	CLP induced-septic ALI mice; orimary PMVECS	Inhibition	ADSCs exosomes relieved inflammation-induced PMVECs ferroptosis and protected lung injury from sepsis via specific delivery of miR-125b-5p and regulation of Keap1	Shen et al. (2023)
		Urolithin A	LPS induced-septic ALI mice; BEAS-2B cells	Inhibition	UA increased the level of antioxidants in lung tissues while reducing LPS-mediated ferroptosis by activating Keap1-Nrf2/HO-1 pathway	Lou et al. (2023)
		Ferulic acid	CLP induced-septic ALI mice; MLE-12 cells	Inhibition	FA treatment inhibited ferroptosis-mediated alveolar epithelial barrier dysfunction in sepsis-ALI via activation of the Nrf2/HO-1 pathway	Tang et al. (2022)
		BACH1	BEAS-2B cells	Inhibition	BACH1 was a novel regulator of LPS-evoked injury through regulation of inflammation response, oxidative stress and ferroptosis via activation Nrf2/HO-1 signaling	Wang et al. (2023b)
	GRP78/PERK/ATF4	RmMANF	CLP induced-septic ALI mice	Inhibition	RmMANF pretreatment attenuates sepsis-associated lung injury by inhibiting the GRP78-dependent PERK/ATF4 pathway and ferroptosis in mice	Zeng et al. (2023)
	ALX/PKA/CREB	PCTR1	LPS induced-septic ALI mice; H1299 cells	Inhibition	PCTR1 potently protects against acute lung injury by inhibiting ferroptosis, which is mediated by ALX/PKA/CREB activation	Lv et al. (2023)
	NCOA4	YAP1	CLP induced-septic ALI mice; MLE-12 cells	Inhibition	YAP1 disrupted the NCOA4–FTH1 reaction and inhibited NCOA4-mediated ferritinophagy to prevent ferroptosis and subsequent mitochondrial ROS-related dysfunction in sepsis-induced ALI.	Zhang et al. (2022)
	STAT3	STING	LPS induced-septic ALI mice; BEAS2B cells	Induction	STING promotes ferroptosis through STAT3 signaling, promoting LPS-induced ALI.	Gu et al. (2024)
	SDC-1/HS	NETs	Patients with sepsis; LPS induced-septic ALI mice; EA.hy926 cells; HULEC-5a cells	Induction	Excessive NETs can damage the endothelial glycocalyx, leading to disruption of the integrity of the SDC-1/HS/HGF complex, thereby obstructing the downstream cMET signaling pathway conduction and inducing endothelial ferroptosis, which further exacerbates tissue injury	Fei et al. (2024)

### 2.3 Role and mechanisms of pyroptosis in sepsis-induced ALI

#### 2.3.1 NF-κB pathway

NF-κB is a dimeric transcription factor in B lymphocytes regarded as a major regulator of inflammation (Li W. et al., 2022). In the classical pathway of pyroptosis, activation of the NLRP3 inflammasome, consisting of NLRP3, ASC, and caspase-1, requires two steps: (I) an initiation step: activation of NF-κB after recognition of PAMPs and DAMPs by pattern recognition receptor, which enhances NLRP3 expression and synthesis; and (II) an activation step: recognition of NLRP3 agonists and inflammasome assembly (Lamkanfi and Dixit, 2014; Huang et al., 2021).

**FIGURE 7:**
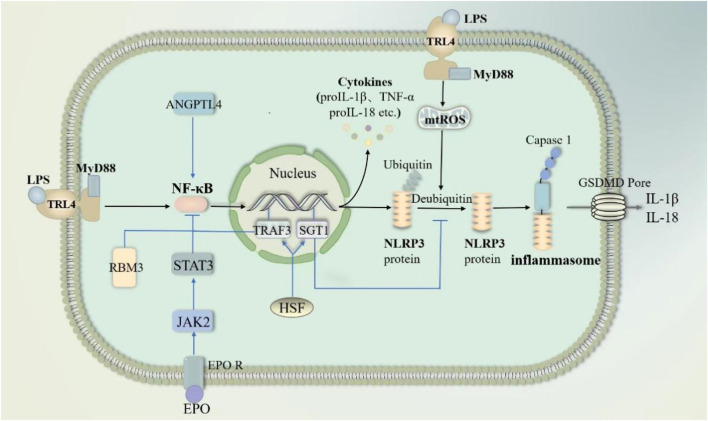
NF-κB pathway through pyroptosis in sepsis-induced acute lung injury. LPS activates nuclear factor kappa-B (NF-κB) through the TRL4/MY88 pathway. NF-κB subsequently activates NLPR3-mediated pyroptosis. Meanwhile, LPS promotes mtROS-induced deubiquitination of NLPR3 proteins to accelerate pyroptosis. Angiopoietin-like 4 enhances NF-κB. In contrast, RNA-binding motif protein 3, erythropoietin and HSF inhibit NF-κB activation. HSF respectively inhibits NF-κB activation and deubiquitination of NLPR3 proteins through TRAF3 and SGT1. Abbreviations: ANGPTL4, Angiopoietin-like 4; RBM3, RNA-binding motif protein 3; EPO, erythropoietin; HSF, heat shock factor; TRAF3, an important class of intracellular signal transduction factors; SGT1, a ubiquitin ligase-associated protein.

NF-κB pathway-mediated “cytokine storm” is one of the major mechanisms of sepsis-induced ALI/ARDS onset and progression (Li W. et al., 2022; Millar et al., 2022). In sepsis, infiltration of the lungs by inflammatory cells such as neutrophils and macrophages is an important part of the worsening of lung injury. Pyropyosis is an influential way for neutrophils to exert their damaging effects. Mouse-specific pyropyosis protein caspase-11 knockout was shown to reduce sepsis-induced neutrophil aggregation, pulmonary edema, and death in mice (Cheng et al., 2017). Macrophage pyropyosis may partially explain uncontrolled lung inflammation in ALI/ARDS (Li et al., 2018; Luo et al., 2021a). LPS in sepsis acts as a PAMP that activates the NK-κB signaling pathway in neutrophils and macrophages via TLR4/MyD88 (Fan and Fan, 2018; Liu and Sun, 2019). The activated NK-κB stimulates the activation of NLRP3 inflammasome, causing pyropyosis of macrophages and neutrophils (Fan and Fan, 2018; Liu and Sun, 2019; Huang et al., 2023). They subsequently release excessive pro-inflammatory cytokines such as IL-1β and IL-18 involved in the development and progression of ALI. In addition, in sepsis, LPS can also activate endothelial cell pyropyosis through the TLR4/MyD88/NF-κB pathway resulting in impaired pulmonary vascular permeability, thereby increasing pulmonary edema (Huang et al., 2023). In sepsis, the endogenous substances cold-inducible RNA-binding protein and angiopoietin-like 4 also activated NF-κB, causing endothelial cell and macrophage pyroptosis, respectively, resulting in ALI (Yang et al., 2016; Sun et al., 2024). Exosomal Tenascin-C binding to TLR4 on macrophages promotes mitochondrial damage through increased ROS production, which activates inflammatory P38/NF-κB pathway, and the DNA damage response triggering macrophage pyroptosis (Gong et al., 2024). However, the expression of some endogenous substances increased. They inhibited pyroptosis by blocking the NF-κB pathway and then attenuated sepsis-induced ALI (Figure 7). For example, heat shock factor 1 can upregulate TRAF3 expression to inhibit the NF-κB pathway and increase SGT1 expression to promote NLRP3 ubiquitination, significantly reducing neutrophil pyropyosis and improving survival in sepsis-induced ALI mice (Shi et al., 2022). The cold shock protein RNA-binding motif protein 3 was elevated in patients with sepsis and mice. It has been demonstrated that RNA-binding motif protein 3 gene deficiency aggravates sepsis-induced ALI through the NF-κB/NLRP3 pathway (Long et al., 2023). Besides erythropoietin would repress NLRP3 inflammasome by deregulating NF-κB p65 phosphorylation and nuclear translocation via the EPOR/JAK2/STAT3/NF-κB axis, which attenuates pulmonary edema and microvascular permeability in mice in sepsis (Cao et al., 2020). In conclusion, Pyroptosis of various lung cells may be one of the mechanisms that induce ALI in sepsis.

For sepsis-induced ALI/ARDS, new pharmacological studies related to the NF-κB pathway in pyroptosis have shown some results. For example, a novel proteolysis-resistant cyclic helix B peptide prevents macrophage pyropyosis by inhibiting NF-κB nuclear translocation to attenuate interstitial stromal edema, intra-alveolar and interstitial hemorrhage in sepsis (Zhang et al., 2020). Loganin significantly regulated macrophage polarization and NLRP3 inflammasome activation, which alleviated alveolar structural damage and inflammatory cell infiltration in a CLP-induced ALI model (Zhang et al., 2021). SYG regulated the TLR4/NF-κB/MAPK signaling pathway to downregulate the expression of pyro-related proteins (including NLRP3, ASC, GSDMD, and caspase 1), thereby reducing the levels of TNF-α, IL-6, and IL-1β (Zhuo et al., 2022).

In summary, pyropyosis of various histiocytes and inflammatory cells in the lungs may be one of the mechanisms that induce ALI in the presence of sepsis. The NF-κB pathway, as a key step in the activation of pyropyotic NLRP3 inflammasome, may be an effective therapeutic target to curb the sepsis-induced cytokine storms that induc ALI.

#### 2.3.2 Nrf2 pathway

In sepsis, large amounts of inflammatory factors can stimulate the release of ROS such as oxygen radicals and hydrogen peroxide from lung-infiltrating macrophages and neutrophils (Guo and Ward, 2007). Oxidative stress in turn exacerbates the release of inflammatory factors in the lungs. These can directly damage alveolar epithelial cells and vascular endothelial cells, affecting alveolar gas exchange and ultimately leading to severe lung dysfunction and structural damage (Guo and Ward, 2007; Sun et al., 2023). Inhibition of pyropyosis may Nrf2 is now found to be an important pathway for exerting a protective effect. Because pyropyosis itself releases massive amounts of inflammatory factors. For example, circVAPA overexpression promotes the Sirt/Nrf2 axis by targeting miR-212-3p, thereby decreasing NLRP3 expression and attenuating sepsis-induced inflammatory damage in ALI (Huang et al., 2024).

ROS upregulates the expression of key substances in pyroptosis (NLRP3, cysteinyl asparaginase-1, IL-1β, and IL-18, etc.) during the initiation step and acts as a second messenger to drive the NLRP3 inflammasome assembly in the activation step (Yu et al., 2017; Zheng et al., 2022). In sepsis, alveolar macrophage pyropyosis can be partially attributed to oxidative stress. An assay indicated that Nrf2-deficient macrophages exhibit elevated levels of cleaved caspase-1, which was attributed to an increase in NLRP3 transcription induced by excessive ROS (Liu X. et al., 2017). IRG1 is a mitochondrial gene. Nrf2/HO-1 expression and anti-inflammatory capacity significantly reduced in IRG1−/− mice receiving CPL, whereas GSDMD-N and serum IL-1β levels significantly increased (Wu et al., 2020; Yang et al., 2023). This phenomenon was alleviated by 4-OI (a derivative of itaconate), which promoted the Nrf2/HO-1 pathway. It manifests as a reduction in pyroptosis of cells and an attenuation of structural damage of lung tissue (Yang et al., 2023). This suggests that the mitochondrial gene IRG1 is a target for Nrf2 to inhibit ALI aggravated by pyropyosis. Endothelial cell pyropyosis under oxidative stress also contributes to the lung structural damage in sepsis. CircEXOC5 is the latest molecule analyzed by gene sequencing to be highly expressed in sepsis-induced ALI (Wang W. et al., 2023). CircEXOC5 can epigenetically inhibit the Nrf2/HO-1 pathway to promote LPS-induced macrophage pyropyosis by recruiting EZH2, which results in interstitial lung edema and alveolar lumen collapse (Wang W. et al., 2023). In contrast, Dihydromyricetin in endothelial cells activated Nrf2 to suppress mtROS-mediated NLRP3 inflammasome activation and subsequent pyroptosis (Hu et al., 2018). In CLP-induced ALI mouse models, citrulline protects lung endothelial cells by activating the Nrf2/ROS/NLRP3 pathway (Xue et al., 2022).

High-mobility group box 1 protein (HMGB1) is a late mediator of endotoxin-induced lethality, resulting in the accumulation of143 inflammatory cells, production of proinflammatory mediators, and pulmonary edema (Yang et al., 2004; Takamiya et al., 2009). Experiments have shown that the Nrf2/HO-1 pathway decreased in HMGB1 release and improves the survival of sepsis-induced ALI mouse models (Tsoyi et al., 2011; Jun et al., 2012; Park et al., 2013). In sepsis, it is released by hepatocytes to deliver extracellular LPS to macrophages and endothelial cells, which directly mediates caspase11 and indirectly mediates caspase1-dependent pyroptosis (Deng et al., 2018). Additionally, the presence HMGB1 in neutrophils, leads neutrophil extracellular traps formation (Xie et al., 2021). Neutrophil extracellular traps act as secondary signals for macrophage activation, stimulating ROS bursts in macrophages and NLRP3 protein deubiquitination (Cui et al., 2023). This promotes the pytoptosis of pulmonary macrophage and exacerbates lung injury.

In general, the Nrf2 pathway in sepsis attenuates pyropyosis-induced massive inflammatory factors and damage to alveolar-capillary structure. The Nrf2 pathway inhibits pyropyosis in sepsis mainly by decreasing the expression or delivery of cell damage factors such as HMGB1 and ROS to alleviate a high amount of inflammatory factors and alveolar-capillary structural damage in sepsis (Table 3).

**TABLE 3:** Mechanisms of pyroptosis in sepsis-induced acute lung injury (ALI).

cell death	Access	Target/compound	Model	Effect	Mechanism	Ref
pyroptosis	Nrf2	Citrulline	LPS-induced ALI mice and MLVECs	Induction	Cit protects pulmonary endothelial cells via activation of the Nrf2 signaling pathway, thereby inhibiting NLRP3 inflammasome activation due to increased levels of intracellular ROS.	Xue et al. (2022)
		gly-pro-ala peptide	CLP-induced ALI mice and J774 cells	Inhibition	Gly-pro-ala peptide inhibited IL-1β secretion, caspase-1 activation, and GSDMD expression in CLP-induced sepsis mice by inhibiting ROS, and alleviated acute lung injury in CLP-induced sepsis mice	Liu et al. (2021c)
		IRG1−/− and 4-OI	IRG1−/− mice and RAW264.7 cells	Induction	The expression of Nrf2/HO-1 significantly in the IRG1−/− mice group increased when receiving 4-OI treatment, and 4-OI significantly inhibited the release of LDH and IL-1β and reduced the expression of GSDMD-N	Yang et al. (2023)
		circVAPA	LPS-induced ALI mice and RAW264.7 cells	Induction	CircVAPA/miR—212—3p/Sirt1 axis also regulates Nrf2 and NLRP3 expression upon LPS challenge. By tar-getting miR—212—3p, circVAPA over—expression negatively regulates the expression of Sirt1 and pyroptosis—related factors (Nrf2 and NLRP3), which alleviates the inflammatory damages in sepsissinduced ALI	Huang et al. (2024)
	MAPK	p38 MAPK	LPS-induced ALI mice, RAW264.7, and NR8383 cells	Inhibition	Inhibiting the p38 MAPK signaling pathway may promote a shift in macrophage cell death from proinflammatory pyroptosis towards a noninflammatory apoptosis process and attenuates acute lung injury	Li et al. (2018)
		CoQ10 and AES	LPS-induced ALI mice	Inhibition	CoQ10 and AES pretreatment prevented LPS-induced ALI via reducing the activation of TLR4/MyD88 signaling to downregulate p38 MAPK and ERK1/2 and then inhibit the NLRP3 inflammasome to protect sepsis-induced ALI	Ali et al. (2021)
		Matrine	CLP-induced ALI mice	Inhibition	Matrine effectively alleviates the symptoms of CLP-induced sepsis in mice, and restrains NLRP3 inflammasome activation by regulating PTPN2/JNK/SREBP2 signaling pathway	Wang et al. (2023d)

#### 2.3.3 miRNAs are involved in sepsis-induced ALI by regulating pyroptosis

**FIGURE 8:**
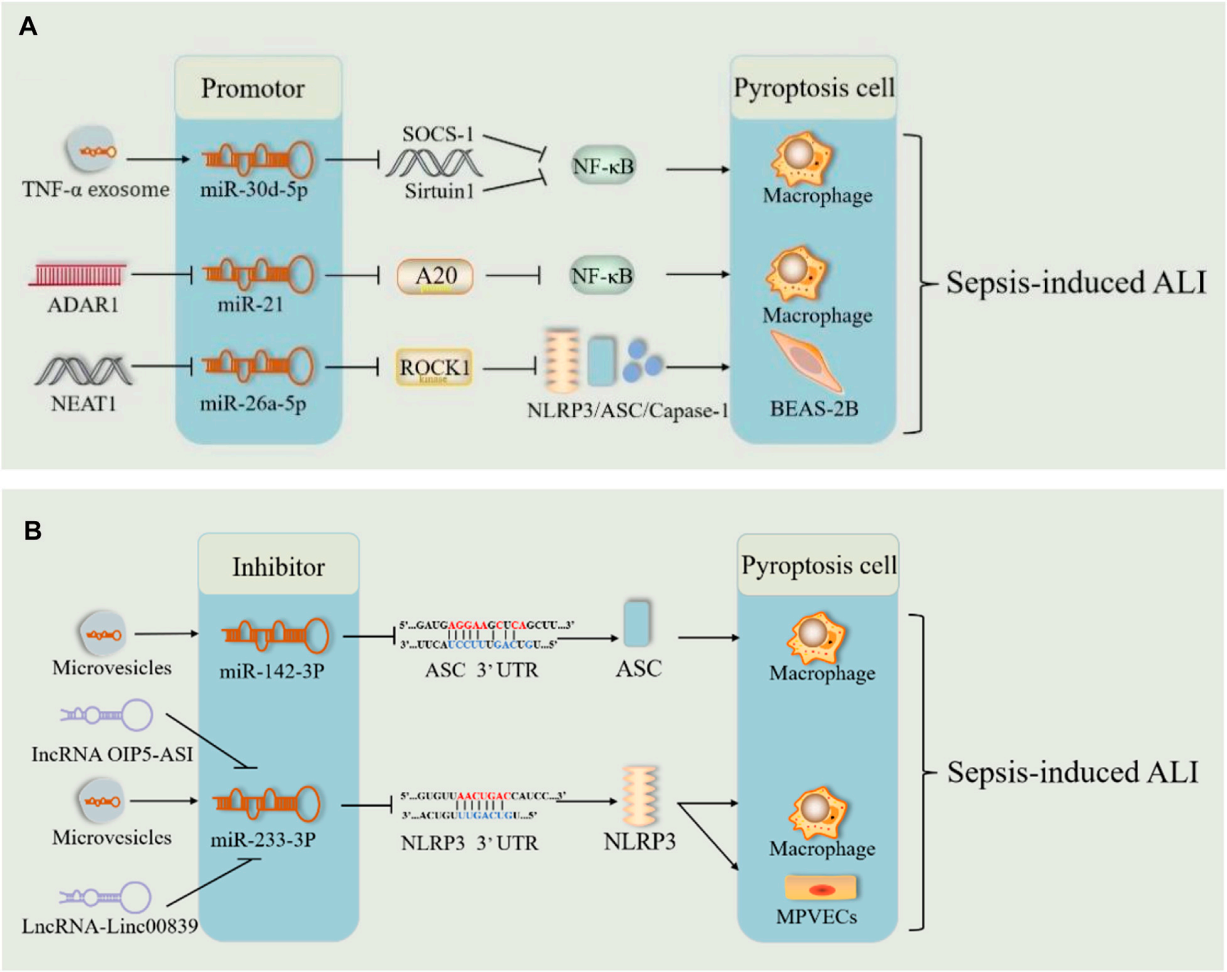
miRNA pathway through pyroptosis in sepsis-induced acute lung injury. **(A)** The microRNA (miR-30Dd-5p/miR-21/miR-26A-5p) pathways play harmful roles in sepsis-induced acute lung injury by promoting pyroptosis. **(B)**. The microRNA (mi142-3p/miR-233-3p) pathways play therapeutic roles in sepsis-induced acute lung injury by inhibiting pyroptosis. Abbreviations: SOCS-1, suppressor of cytokine signaling; ADAR1, poly (adenosine diphosphate-ribose) polymerase–1; A20, an anti-inflammatory protein; NAT10, N-acetyltransferase; MPVECs, mouse pulmonary microvascular endothelial cells; 3′UTR, 3′untranslated regions of mRNA.

miRNAs have emerged as key players in sepsis. Previously, we mentioned that miRNAs can regulate sepsis-induced ALI via cellular autophagy. Song et al. reported for the first time that miR-34a enhances lung tissue injury by promoting pyroptosis in mice with sepsis-induced ALI (Chen et al., 2020). In sepsis, TNF-α stimulated neutrophils to produce exosomes carrying miR-30d-5p, targeting and stimulating M1 macrophage activation and pyroptosis (Jiao et al., 2021) (Figure 8A). ADAR1, which is significantly reduced in polymorphonuclear neutrophils from patients with sepsis, reduced the expression levels of NLRP3, caspase1, GSDMD, and Il-1β via the miR-21/A20/NLRP3 axis and suggested that miR-21 is a facilitator of ALI in sepsis (Zhao et al., 2024) (Figure 8A). These results indicated that miRNAs may be responsible for ALI via pyroptosis in sepsis. However, miR-135b-5p inhibits cell pyroptosis by downregulating GSDMD, alleviating sepsis-induced ALI (Zang et al., 2022). This suggests that miRNAs involved in sepsis may also protect against ALI by inhibiting pyroptosis. In patients with severe sepsis, downregulation of miR-223 may induce M1 polarization, whereas in mice with LPS-induced sepsis, administration of miR-223 can attenuate sepsis by promoting M2 polarization (Dang and Leelahavanichkul, 2020). During pulmonary inflammation, neutrophil activation increases miR-223 release via microvesicles, which subsequently targets PARP-1 in alveolar epithelial cells to exert a protective effect (Neudecker et al., 2017) (Figure 8B). Intracellular miR-223/142 suppresses the lung inflammasome by synergistically inhibiting NLRP3 and ASC to inhibit the activation of the NLRP3 inflammasome in macrophages (Xiong et al., 2023) (Figure 8B). The lncRNA OIP5-AS1 aggravated LPS-induced ALI/ARDS by causing lung capillary endothelial cell injury via the miR-223/NLRP3 axis (Ji et al., 2022) (Figure 8B). Fu et al. demonstrated both *in vivo* and *in vitro* that sevoflurane improved improves pulmonary endothelial cell pyropyosis and alveolar septal edema thickening in sepsis-induced ALI via a novel lncRNA (LINC00839)/miR-223/NLRP3 axis (Fu et al., 2022b) (Figure 8B). Therefore, miR-223 may serve as a new therapeutic target for sepsis-induced ALI. However, further *in vitro* and *in vivo* experiments are needed for validation.

Additionally, lncRNAs act as “molecular sponges” for miRNAs, competitively binding to miRNA response elements and affecting miRNA-induced gene silencing, thereby upregulating the mRNA expression of target genes (Huang, 2018; Tang et al., 2021). The lncRNA-miRNA-mRNA axis regulates sepsis-induced ALI by modulating the levels of inflammatory factors and apoptosis (Tang et al., 2021). NEAT1 (lncRNA) is known to be upregulated in sepsis. It exacerbates sepsis-induced ALI by enhancing pyroptosis through the repression of the miR-26a-5p/ROCK1 axis (Fan et al., 2023) (Figure 8A). Similarly, lncRNA4 344 promoted pyroptosis in the inflammatory response to intense LPS-induced ALI by inhibiting miRNA-138-5p/mRNA NLRP3 (Luo et al., 2021b). miR-233 also exerted a role in sepsis-induced ALI through the lncRNA-miRNA-mRNA axis. Thus, the lncRNA-miRNA-mRNA axis modulates sepsis-induced ALI by regulating pyroptosis. miRNA-mediated pyroptosis has a dual role in sepsis-induced ALI. Further studies are required to determine the role of the lncRNA-miRNA-mRNA axis in pyroptosis and the inflammatory cascade response.

#### 2.3.4 MAPK pathway

MAPK is a serine/threonine protein kinase widely abundant in eukaryotic cells that phosphorylates target proteins through a three-tiered kinase cascade pathway to transactivate cells and their nuclei (Li W. et al., 2022). MAPK consists of four subgroups: extracellular signal-regulated kinases 1 and 2 (ERK1/2), c-Jun amino-terminal kinases 1–3 (JNK1-3), p38, and ERK5 (Cargnello and Roux, 2011). In sepsis, the activated MAPK pathway increases the release of IL-6, IL-2, TNF-α, and other factors from inflammatory cells, such as macrophages, causing histopathologic changes in lung tissue and increased pulmonary vascular permeability (Li W. et al., 2022). Studies have demonstrated that the inhibition of the MAPK pathway is protective in sepsis-induced ALI (Liao et al., 2023). The p38MAPK-specific inhibitor SB203580 blocked the secretion of key substances for pyroptosis (NLRP3, caspase-1, and IL-1β, among others) and other inflammatory factors in macrophages in ALI models (Li et al., 2018). Notably, hyperphosphorylation of MAPK molecules could activate the transcription factor NF-κB and the ensuing inflammatory response (Liu et al., 2020). SB203580 pre-administration inhibits LPS-induced NF-κB phosphorylation and attenuates inflammatory responses such as neutrophil infiltration and lung damage (Kim et al., 2006). Moreover, if TLR4/MyD88 signaling, which causes NF-κB activation, is downregulated, it may also suppress NLRP3 inflammasome activation via the p38 MAPK and ERK1/2 pathways (Ali et al., 2021). ERK1/2 and p38 are involved in cell pyroptosis during sepsis. They may also interact with the NF-κB pathway, leading to the development of ALI. JNK is an essential kinase activated during pyroptosis. This kinase triggers NLRP3 phosphorylation. Notably, phosphorylation of NLRP3 at S194 is an integral initiating event in inflammasome activation (Song N. et al., 2017). Deubiquitination of NLRP3 is required for its activation, and S194 phosphorylation of NLRP3 is a crucial regulator of NLRP3 activation upstream of deubiquitination (Juliana et al., 2012; Song N. et al., 2017). In addition, matrine inhibited macrophage pyroptosis via the PTPN2/JNK/SREBP2 pathway by inhibiting ASC phosphorylation and negatively regulating NLRP3 inflammasome activation (Wang X. et al., 2023). These results suggest that the MAPK pathway promotes ALI by activating pyroptosis in sepsis and could be a therapeutic target for research on acute lung injury in sepsis (Table 3).

Previous studies have shown that pyroptosis is closely associated with the development, severity, and prognosis of ALI by sepsis. Although pyroptosis has a two-sided nature in sepsis-related organ injury, this study mainly focused on the role of pyroptosis in sepsis-related lung tissue injury. The indirect or direct inhibition of pyroptosis-associated proteins through various pathways has been shown to exert protective effects against sepsis-induced acute lung injury.

### 2.4 The crosstalk between various programmed deaths in sepsis induced ALI

Increasing evidence suggests that there is extensive crosstalk between various types of programmed cell death, key triggers, effectors, and effective drugs for the progression of ALI in sepsis. mTOR, a hotspot for negative autophagy regulators (Saxton and Sabatini, 2017), plays a role in ferroptosis and apoptosis. It has been demonstrated that mTOR can influence the onset of iron death by mediating the accumulation of polyunsaturated fatty acids and can also increase cellular resistance to ferroptosis via monounsaturated fatty acids (Xl et al., 2022). Ketamine inhibits the mTOR pathway by promoting AMPK overexpression, thus promoting autophagy, reducing apoptosis, and attenuating sepsis-induced lung injury in mice (Cao et al., 2022). These studies suggest that mTOR plays a protective role in sepsis-induced ALI by regulating multiple cell death mechanisms. However, the role of mTOR in sepsis-induced ALI requires further investigations. As previously mentioned, JNK plays a key role in pyroptosis activation. However, inhibition of JNK activity can promote autophagy and alleviate lung injury (Zheng et al., 2020). Different miRNAs may play different roles in sepsis-induced ALI; in many cases, one miRNA regulates multiple modes of death. For example,: microRNA-34a can inhibit autophagosome formation by binding to the untranslated region of ATG4B, and while inhibiting autophagy, it increases oxidative stress and pyroptosis to aggravate septic ALI (Chen et al., 2020). Luo et al. demonstrated in their experiments that the inhibition of miRNA-138-5p overexpression attenuated NLRP3-mediated pyroptosis in the inflammatory response to ALI (Luo et al., 2021b). The autophagy-induced reduction of cytoplasmic mtDNA levels via methylation of the miR-138-5p promoter increased miR-138-5p expression levels, which subsequently attenuated the activation of the NLRP3 inflammasome and focal death of AMs during sepsis-induced ALI (Liu et al., 2023).

Several drugs can improve sepsis-induced ALI by simultaneously modulating different types of cell death. GYY4137(H_2_S) inhibits the NLRP3 inflammasome by decreasing xanthine oxidase activity and mitochondrial ROS production (Castelblanco et al., 2018). Li et al. later pointed out that GYY4137 can significantly inhibit CLP-induced ALI, and the regulation of PDGFRβ/Akt/NF-κB/NLRP3 signaling pathway is the central mechanism of its anti-inflammatory effect (Li J. et al., 2021). Reportedly, it is proposed that H_2_S alleviates iron death by blocking mTOR signaling and inhibiting autophagy to alleviate sepsis-induced ALI (Li J. et al., 2022). Resveratrol can reduce LPS-induced apoptosis in MH-S cells by activating the VEGF-B signaling pathway to alter Bax/Bcl-2 imbalance and inhibit LPS-induced autophagy, thereby attenuating ALI in sepsis (Yang et al., 2018). Vitamin K2 reduces lung apoptosis by inhibiting LPS-induced activation of P38 MAPK and LPS-induced elevation of HO-1 to inhibit iron death and achieve a protective effect against lung injury (Wang Y. et al., 2023). The progression of sepsis-induced ALI results from interactions between various types of programmed cell deaths. Different types of programmed cell death can either synergistically promote or constrain each other. The existence of common key initiators and effectors in various types of programmed cell death provides insights for future drug research and improvement of therapeutic efficacy.

## 3 Limitation

Most current experiments are based on animal and cellular models, yet whether sepsis-induced acute lung injury in humans exhibits pathophysiologic processes that are essentially similar to those in cells alone or in mice cannot be guaranteed. For example, the hallmark proteins of pyropyosis are not identical in humans and mice, and the pathways of pyropyosis they mediate are not entirely the same. Moreover, for some substances that are released in mice with sepsis-induced acute lung injury may not be present in humans. Therefore, the applicability and effectiveness of the above signaling pathways and related drugs in humans remains to be investigated. In terms of autophagy, does simply targeting autophagy offer more advantages than disadvantages when treating patients with sepsis? Additionally, identifying the optimal timing for promoting or inhibiting autophagy as a protective measure requires further investigation since incorrect timing of treatment may accelerate disease progression.

In some signaling pathways, although it can be demonstrated that an upstream factor can influence the production of a downstream factor, more complex or critical signaling processes may be involved, which have not been studied in great detail.

Although articles on the role of autophagy, ferroptosis, and pyropyosis in sepsis induced-ALI mice through multiple signaling pathways, respectively, have been included, there are still other articles on signaling pathways, as well as other types of cell death that have not been covered in this review. As a result, the literature covered in this review is not comprehensive enough.

## 4 Future perspectives

Based on the above signaling pathways, future articles can make more detailed supplementary studies to clarify the key links of signal transduction and the mechanism of interactions between substances. The more detailed preclinical studies will provide a reliable pharmacological basis for the development of safer and more effective new drugs.

It is important to note that human function is a complex and integrated system. More clinical studies are needed to confirm whether the appeal signaling pathways still play the same role in sepsis-induced acute lung injury in humans, whether there are differences in the intermediate steps, and whether they still play an important role. There is an even greater need to determine the toxicity, safety, side effects, and effective dose of various drugs and substances in humans.

The mutual crosstalk between the various deaths should serve as an important research direction for the occurrence, development and prognosis of acute lung injury in sepsis. Various types of deaths can both promote and inhibit each other, and maintaining a balance between them may play a positive role. However, preclinical and clinical studies in these areas are currently lacking.

In addition, how to control the timing nodes between the early protective and subsequent destructive effects of various cell death types requires further research. These could prevent both suppressing their protective effects too early and delaying the time to actively target therapy.

## 5 Conclusion

Many studies have suggested that sepsis-induced ALI can be attenuated by regulating autophagy, ferroptosis, and pyroptosis, mainly by focusing on the pathways and targets related to sepsis-induced ALI. Based on existing articles, this review summarizes the main pathways in the current study: AMPK, PI3K/Akt/mTOR, mtDNA, miRNA, Xc–GSH-GPX4, Nrf2, P53, ACSL4, NF-κB, MAPK and other signaling pathways. Recent studies have mainly focused on the theoretical aspects of the regulatory mechanisms of these pathways. However, clinical research on targeted therapies is still in the early stages of development. In addition, sepsis-induced ALI may be associated with multiple cell death processes, including other modes of cell death not mentioned in this review, such as apoptosis, cuproptosis, and cell necrosis. Therefore, more animal experiments and clinically relevant studies are required to understand better the roles of different types of cell death in sepsis-induced ALI. Future studies should focus on the role of crosstalk between cell death and sepsis-induced ALI to provide new clinical treatments for sepsis-induced ALI.
